# Supplementary figures and images for: Supervised learning using routine surveillance data improves outbreak detection of Salmonella and Campylobacter infections in Germany
Source: PLoS One. 2022 May 5;17(5):e0267510. doi: 10.1371/journal.pone.0267510 (PMC9070876; doi:10.1371/journal.pone.0267510)

A

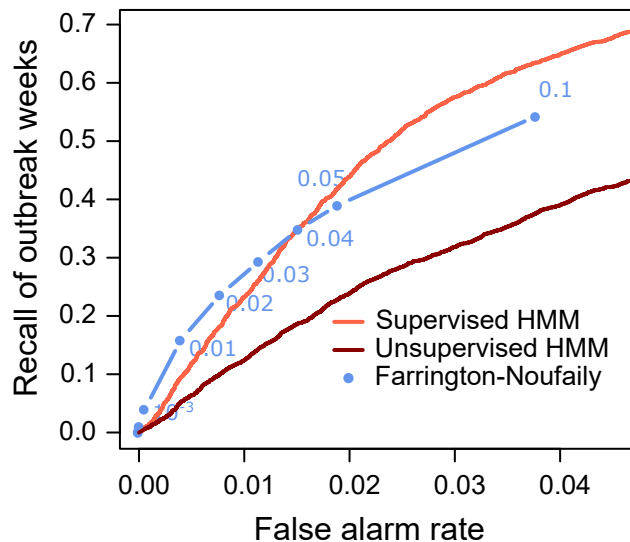

B

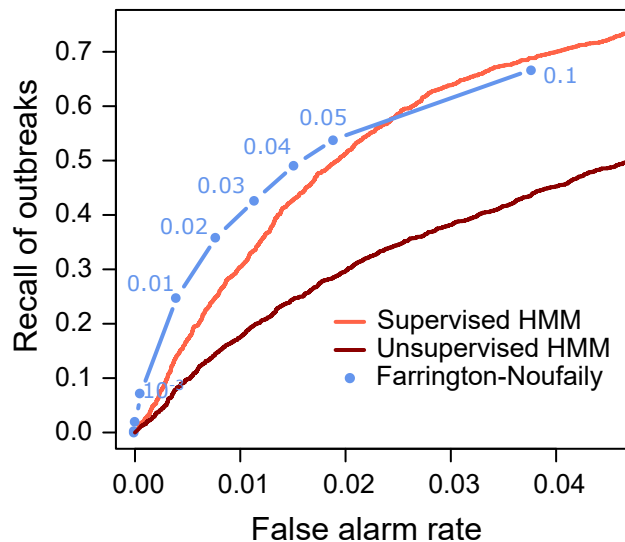

C

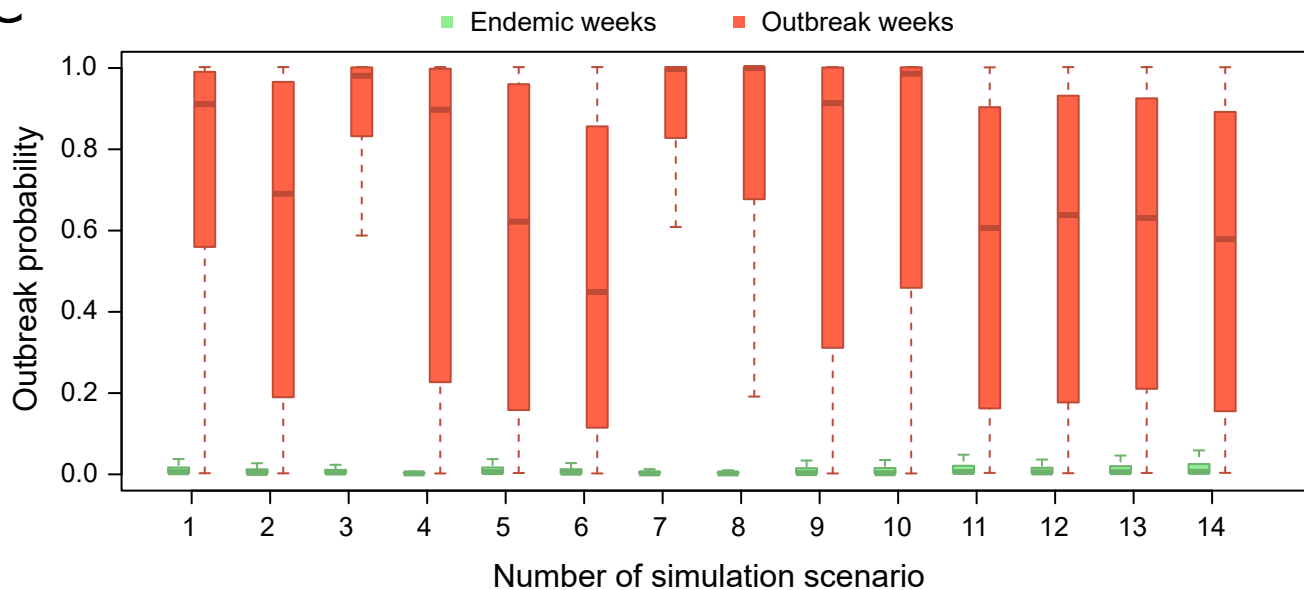

Supplement: S1 Fig — (A) ROC curve showing the false alarm rate and recall of oubtreak weeks for all 14 simulation scenarios. (B) ROC curve showing the false alarm rate and recall of oubtreaks for all 14 simulation scenarios. (C) Boxplots of posterior probabilities of known endemic (green) and outbreak (red) weeks for all 14 simulation scenarios. (PDF) [file pone.0267510.s001.pdf]

# Salmonella

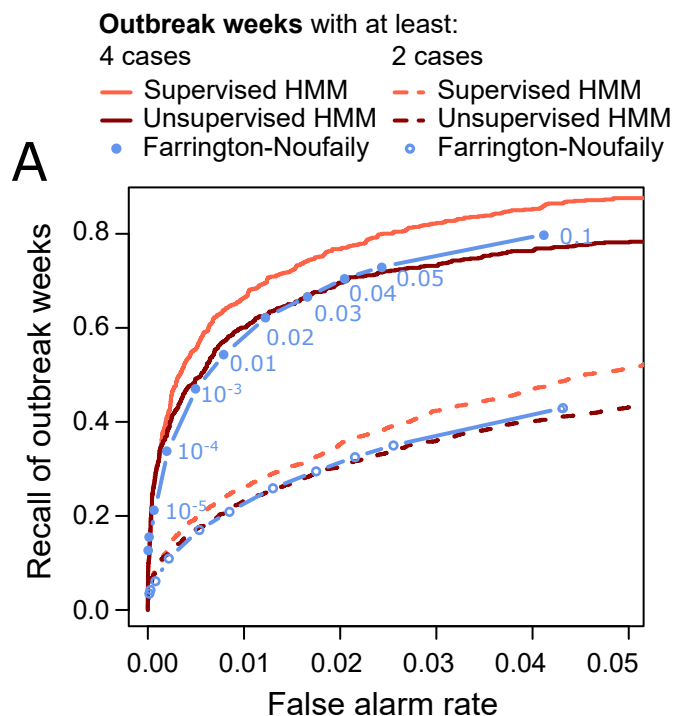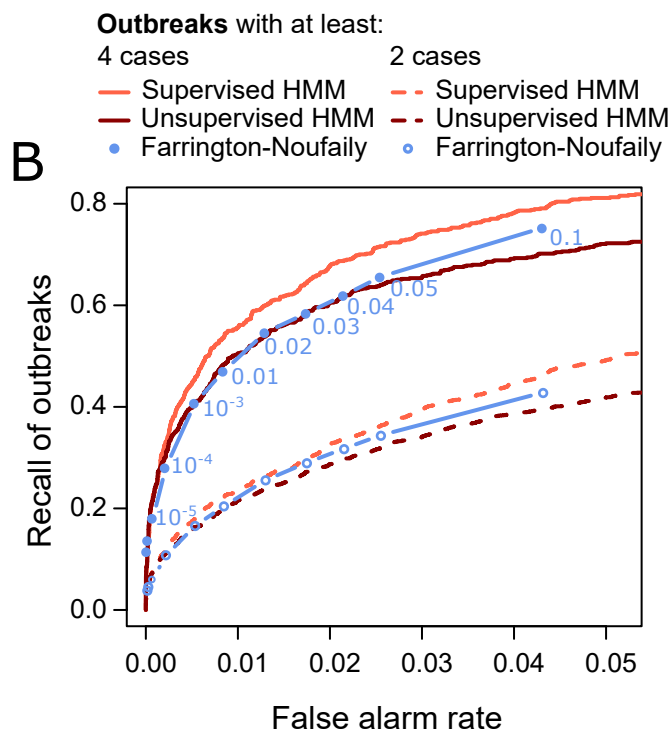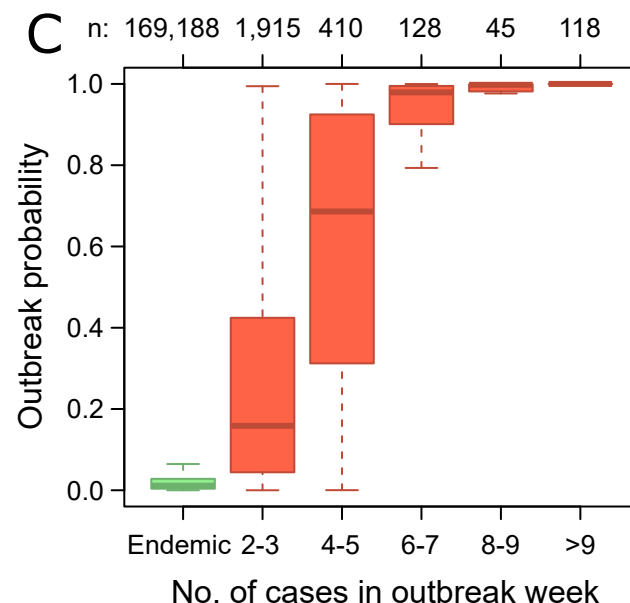

# Campylobacter

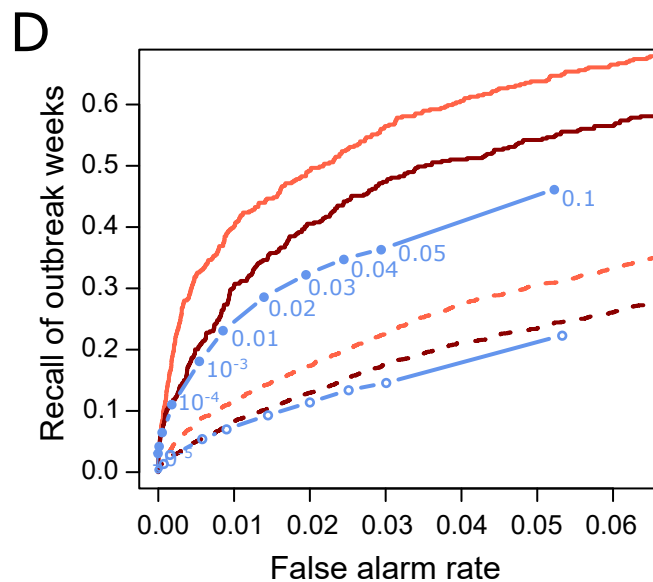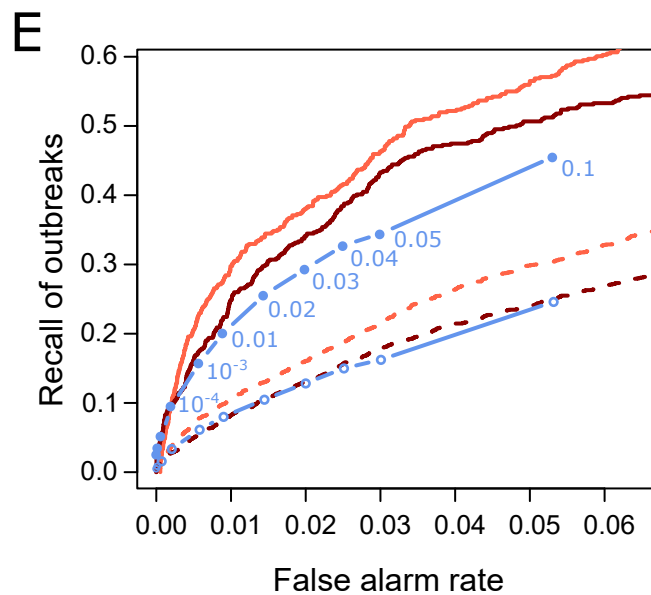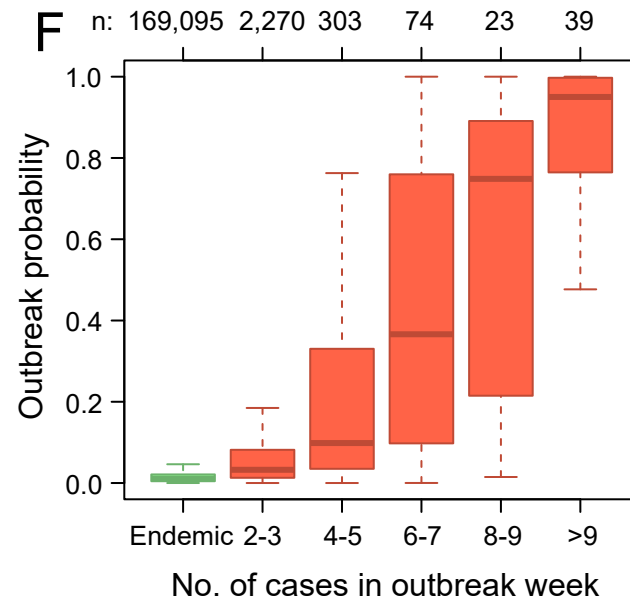

Supplement: S2 Fig — (A) ROC curve showing the false alarm rate and recall of oubtreak weeks for the Salmonella data. Performance was evaluated with outbreaks that involved at least two or four cases. The FN alogrithm was applied with cutoffs 10−6, 10−5, 10−4, 0.001, 0.005 and 0.01 using threshold method ‘nbPlugin’. The HMM was applied with the negative Binomial distribution. (B) The same as (A) but showing the recall of oubtreaks. (C) Boxplots of oubtreak probabilities form the HMM are shown for known endemic (green) and outbreak (red) weeks. Outbreaks were further divided by their size (i.e. the number of cases reported in an outbreak per week). (D-F) The same as (A-C) for Camplyobacter data. (PDF) [file pone.0267510.s002.pdf]

**A**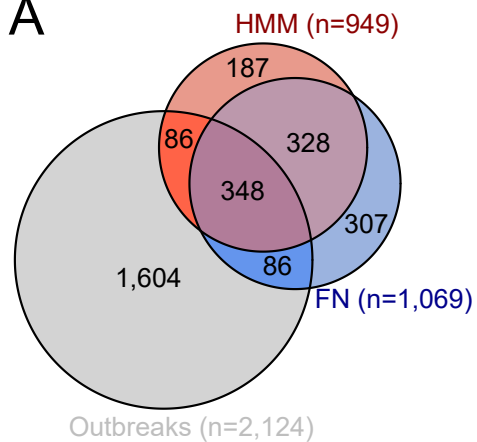**B**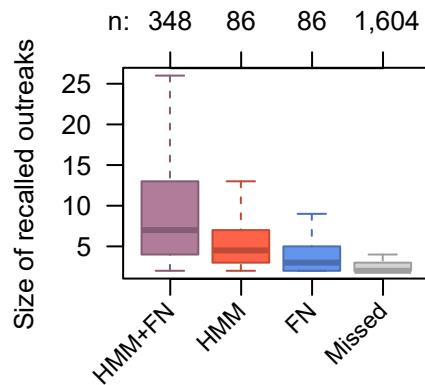**C**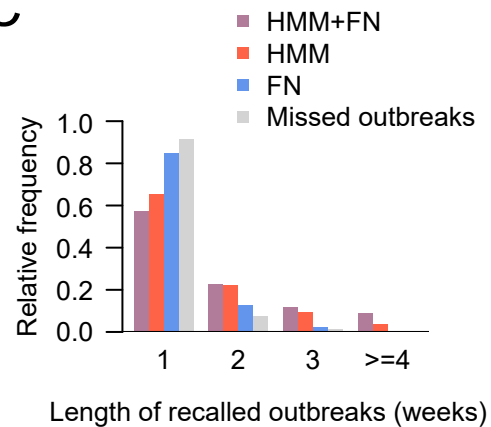**D**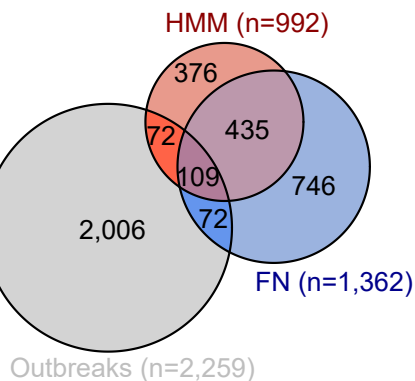**E**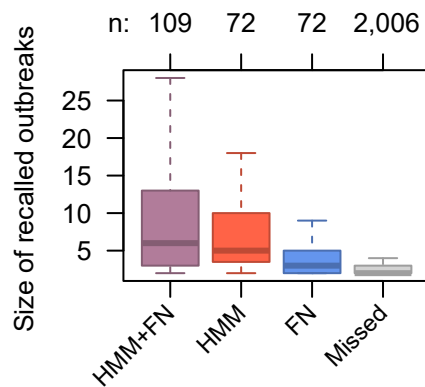**F**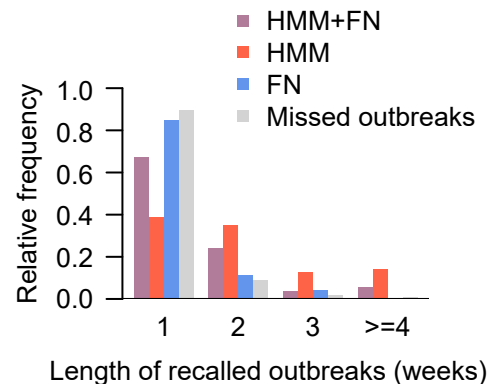

Supplement: S3 Fig — (A) Venn diagram showing the overlap between outbreaks recalled by both methods. TheFN algorithm was applied with cutoff 0.01 using threshold method ‘nbPlugin’. The cutoff for the HMM outbreak probability was chosen to get the same recall as the FN algorithm. (B) The number of cases in outbreaks recalled by both, one or none of the applied methods. (C) Distribution of the duration (in weeks) of outbreaks recalled by both, one or none of the applied methods. (D-F) The same as (A-C) for the Campylobacter data. (PDF) [file pone.0267510.s003.pdf]
