# Supplementary material for: Supervised learning using routine surveillance data improves outbreak detection of Salmonella and Campylobacter infections in Germany
Source: PLoS One. 2022 May 5;17(5):e0267510. doi: 10.1371/journal.pone.0267510 (PMC9070876; doi:10.1371/journal.pone.0267510)
Supplement: S1 Table — Parameters for all 14 simulation scenarios are shown. (DOCX) [file pone.0267510.s005.docx]

| Scenario | β 0 | β 1 | β 2 | β 3 | φ |
| --- | --- | --- | --- | --- | --- |
| 1 | 0.1 | 0 | 0.6 | 0.6 | 1.5 |
| 2 | 0.1 | 0.0025 | 0.6 | 0.6 | 1.5 |
| 3 | -2 | 0 | 0.1 | 0.3 | 2 |
| 4 | -2 | 0.005 | 0.1 | 0.3 | 2 |
| 5 | 1.5 | 0 | 0.2 | -0.4 | 1 |
| 6 | 1.5 | 0.003 | 0.2 | -0.4 | 1 |
| 7 | 0.5 | 0 | 0.5 | 0.5 | 5 |
| 8 | 0.5 | 0.002 | 0.5 | 0.5 | 5 |
| 9 | 2.5 | 0 | 1 | 0.1 | 3 |
| 10 | 2.5 | 0.001 | 1 | 0.1 | 3 |
| 11 | 3.75 | 0 | 0.1 | -0.1 | 1.1 |
| 12 | 3.75 | 0.001 | 0.1 | -0.1 | 1.1 |
| 13 | 5 | 0 | 0.05 | 0.01 | 1.2 |
| 14 | 5 | 0.0001 | 0.05 | 0.01 | 1.2 |
