# Supplementary material for: Supervised learning using routine surveillance data improves outbreak detection of Salmonella and Campylobacter infections in Germany
Source: PLoS One. 2022 May 5;17(5):e0267510. doi: 10.1371/journal.pone.0267510 (PMC9070876; doi:10.1371/journal.pone.0267510)
Supplement: Attachment — Submitted filename: point_to_point_response.pdf [file pone.0267510.s006.pdf]

Point-to-point response to Reviewers' comments:

Reviewer #1:

Overall, I think that it presents a fair assessment regarding the question of whether using past outbreaks to allow supervised learning has the potential to outperform unsupervised learning which is the main purpose of the work.

The conclusion "...our results are promising that leveraging outbreak data with supervised learning will improve disease outbreak detection." seems fair although the will might be softened to may.

Response: We agree and changed it to "may".

I don't think that the approach as presented offers a reason to change to this for those currently deploying other algorithms - and this is not claimed by the authors. More generally, in common with the comparator approaches, the fact of an outbreak being probable is returned but this is a limited benefit. Although done a lot, this is very limited information and, for example, doesn't make clear which cases belong to the outbreak and which do not. In practice the capacity of genetic sequencing and analysis to accurately detect and characterise outbreaks in Salmonella in particular makes such approaches largely redundant for this use case. The authors make a fair hand at noting that what is in practice detected is limited.

Response: We agree that genetic sequencing is a great tool to characterize disease outbreak and overall the information our algorithm gives is limited. However, genomic Sequencing is not yet applied in an exhaustive manner. Genomic Sequencing might provide an accurate picture of an outbreak, but cases are also usually reported before genomic sequences are available and thus the surveillance of these time series might detect outbreaks earlier, which is important to prepare and implement public health measures in a timely manner.

One technical issue is that modelling an outbreak as multiplicative relative to background rates appears strange. A secular trend of increasing incidence or a seasonal peak period would then need a larger outbreak to be detectable than when baseline levels are low. An additive model for the outbreak term might be a better fit. This approach was also applied to the simulation with outbreaks size proportional to the route of the variance of weekly counts such that the signal to be detected also carried this unlikely premise. The discussion might consider this choice in simulation and analysis and the alternative of non-multiplicative relationships.

Response: In the time series of infectious disease case counts, the variance usually increases with the background rates and thus the detection of small outbreaks becomes more difficult with increasing background, also when using an additive model. However, if the background rates are high, the relevance of small outbreak for public health surveillance and infection control is limited, because small outbreaks will not contribute much to the overall occurrence of infection. Nonetheless, we agree that this is a limitation of our model and we now mention this in the discussion.

The idea of seeking what is special about an outbreak vs modelling aberration from normal is appealing conceptually. I would have thought it might end up performing equivalently mathematically but the authors findings suggest that it does not and may be better in practice as well as a better theoretical fit as per their conclusion pasted in above.

Reviewer #2:

Zacher and Czogiel propose a supervised HMM method for detecting potential disease outbreaks in Germany. This method takes advantage of the routinely collected outbreak data as known hidden states for improving detection performance. The effectiveness of this method was verified in experiments. The manuscript was well written. This paper will be ready for publication if the following minor problem is addressed.

Page 1, line 17: "on par or better than" --> "on par with or better than"

Response: Changed.
